# Laparoscopic treatment of a patent *ductus venosus* and the use of indocyanine green to monitor perioperative hepatic function

**DOI:** 10.1093/jscr/rjy026

**Published:** 2018-02-22

**Authors:** Marcos V Perini, Graham M Starkey, Su Kah Goh, Georgina E Riddiough, Christopher Christophi

**Affiliations:** 1 Hepato-pancreato-biliary and Transplantation Unit, Austin Health, Melbourne, Australia; 2 Department of Surgery, Austin Health, The University of Melbourne, Melbourne, Australia

## Abstract

Patent *ductus venosus* (PDV) is an uncommon but important congenital portocaval shunt that can lead to numerous complications if untreated. This case describes the successful management of a 17-year-old male with symptomatic PDV. Doppler ultrasonography and contrast-enhanced computed tomography (CT) confirmed a large communication between the left portal vein and the inferior vena cava. Angiography demonstrated a large and high flow PDV which precluded its therapeutic embolization. Based on these findings, laparoscopic closure of the PDV was elected and successfully performed. Perioperative indocyanine green (ICG) clearance was performed and marked improvement was observed following the occlusion of the PDV. The patient showed immediate resolution of symptoms post-operatively and remains asymptomatic 2 years after his surgery. Laparoscopic approach to the management of PDV is feasible. ICG clearance, for the first time, was demonstrated in this setting to be a useful and rapid bedside test for the real-time assessment of liver function.

## INTRODUCTION

The *ductus venosus*(DV) arises from the posterior aspect of the left portal vein and passes superolaterally to join the left hepatic vein. During foetal development, the DV shunts oxygenated blood away from the liver. Complete closure normally occurs 17 days after birth [1]. The remnant ligamentum venosum becomes fibrotic and lies within the fissure separating the caudate and the left lobe of the liver. This structure that leads to the left hepatic vein is also a critical landmark in split liver transplantation [2].

Complications of a Patent DV (PDV) include encephalopathy, fatty infiltration of the liver, focal nodular hyperplasia and hepatopulmonary syndrome [3]. Taking into the account of these factors, the placement of an endoluminal occlusive stent or even surgical occlusion (ligation/division/stapling) of the shunt can be attempted to minimize the complications of PDV [4].

The evaluation of hepatic function can be performed by comparing serum ammonia levels and liver biochemistry before and after the procedure. However, such tests cannot be readily performed intra-operatively. Indocyanine green (ICG) is a non-toxic fluorescent dye that is exclusively excreted by the liver [5]. The rate of which ICG is removed from the circulation after intravenous injection enables the quantification of hepatic function. ICG clearance is measured as plasma disappearance rate (PDR) and retention after 15 min (R15). The rapid and non-invasive nature of using ICG to quantify hepatic function has attracted significant interests in many aspects of hepatic surgery [6, 7]. Its current role in the assessment of shunts like PDV is unclear and has not been reported.

Here, we describe the second case in the English literature of laparoscopic treatment of PDV and the first case in which intra-operative assessment of liver function with ICG was performed.

## CASE REPORT

A 17-year-old male patient was referred for the assessment of hepatic encephalopathy associated with fine tremors, somnolence and attention deficit on a background of recently diagnosed PDV.

**Figure 1:**
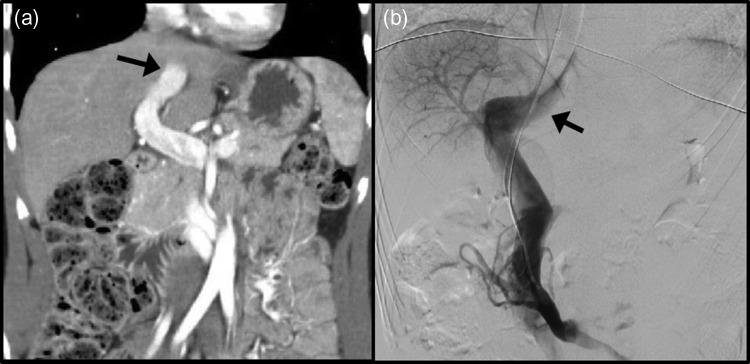
Radiological assessment of the patent *ductus venosus* (PDV) by (**a**) computed tomography and (**b**) digital subtraction angiography. Black arrow: PDV traversing towards the inferior vena cava.

**Figure 2:**
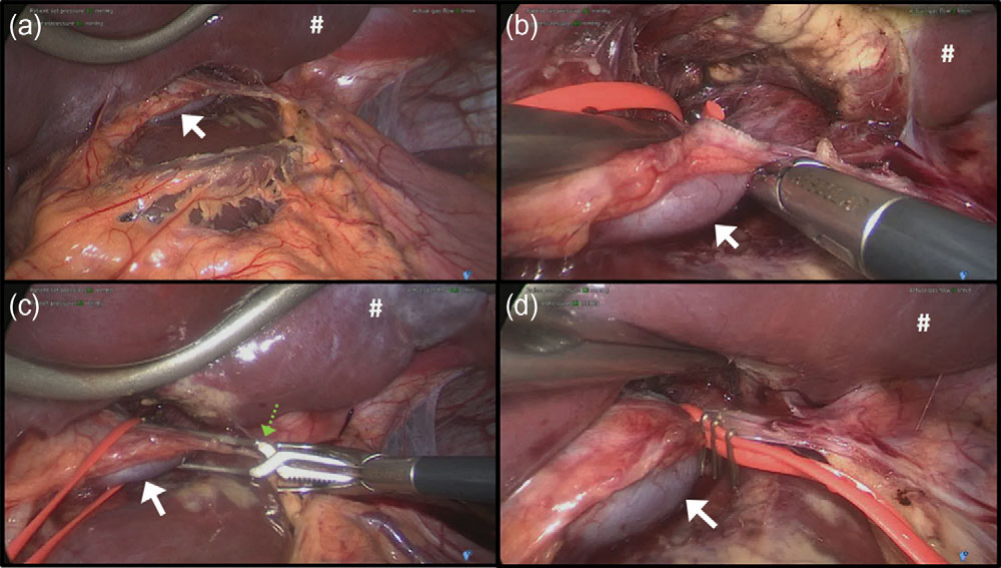
Laparoscopic view of (**a**) gastro-hepatic ligament, (**b**) patent *ductus venosus* (PDV), (**c**) loop encircling the PDV with a vascular clamp occluding the PDV and (**d**) partial occlusion of the PDV with tightened vessel loop secured with surgical clips. White arrow, PDV; green arrow, surgical clamp used for temporary occlusion of the PDV; #, retracted left lobe of the liver.

Pre-operative blood tests showed normal liver biochemistry and post-prandial hyperammonaenia (162 μmol/L: normal 16–60 μmol/L). Doppler ultrasound and computed tomography (CT) demonstrated a large communication between the left portal vein and the left hepatic vein with a patent and hypoplastic portal bed (Fig. 1a). Owing to the calibre and flow of the shunt, endovascular occlusion was not feasible (Fig. 2). Surgical treatment by a two-stage laparoscopic approach (partial occlusion followed by division) was subsequently planned. Pre-operative ICG clearance was measured as per institutional protocol (PDR: 5.5% min^−1^ and R15:43.8%) [6].

Ports were placed as followed: 10 mm port at the umbilicus, 10 mm port in the left mid anterior clavicular line, 5 mm in the right upper quadrant and 5 mm incision in the sub-xyphoid region for the Nathanson liver retractor (Cook Medical, USA).

The liver was normal with no evidence of portal hypertension. Laparoscopic intra-operative ultrasound (IOUS) showed a patent portal vasculature. The gastro-hepatic ligament was divided close to the liver to expose the caudate lobe and the PDV (Fig. 2a). The PDV was subsequently encircled with a vessel loop (Fig. 2b).

The PDV was temporarily clamped and IOUS was performed to assess the intra-hepatic portal vasculature (Fig. 2c). The portal vasculature remained patent and the splanchnic vasculature did not demonstrate acute portal hypertension. ICG clearance was repeated (PDR: 11.9% min^−1^ and R15: 16.8%).

The decision was undertaken to partially occlude the PDV to minimize the risks of mesenteric congestion or portal venous thrombosis. The vessel loop was tightened and secured with surgical clips and left *in-situ*, with a view to divide the PDV at a later stage (Fig. 2d). After partial occlusion, ICG clearance was repeated (PDR: 19.5% min^−1^ and R15: 5.4%).

Day 1 post-operatively, the patient remained well and his symptoms continued to improve. His ammonia levels normalized to 43 μmol/L. ICG clearance was assessed (PDR: 14.2% min^−1^ and R15: 11.9%). Doppler ultrasound confirmed portal venous patency and a completely occluded PDV. In view of this finding, therapeutic anticoagulation was initiated to minimize the risks of portal vein thrombosis. The patient was discharged home after 3 days.

At 3 months, a quad-phase CT of the liver demonstrated an occluded PDV with patent extra- and intra-hepatic portal vasculature. An increase in liver volume from 830 to 1468 mL was also observed on CT volumetry analysis. Anticoagulation was ceased. The patient remains asymptomatic at 24 months. Based on these findings, the initial plan to divide the PDV at a later stage was no longer required.

## DISCUSSION

Reported treatments of PDV include radiological occlusion, surgical ligation and liver transplantation. The use of these procedures, to date, remains heterogeneous owing to the rarity of the condition [8].

Shunt occlusion is the treatment of choice for PDV and this can be performed through an endovascular or a surgical approach. A key complication that can occur post-operatively is portal venous thrombosis. This is critical in patients with hypoplastic intra-hepatic portal vasculature, whereby the sudden occlusion of the shunt can lead acute portal hypertension, gastrointestinal bleeding and intestinal infarct [8].

Laparoscopic treatment of other porto-systemic shunts has been reported [9]. However, only one case of PDV occlusion by a laparoscopic approach has been reported, of which the patient developed portal venous thrombosis that required thrombolysis [10].

In order to minimize the risks of complications in this case, a two-stage procedure was initially planned to partially occlude the shunt first, and divide the shunt at a later stage. It was postulated that this will allow the portal vasculature to adapt to the increased portal flow changes. This manoeuvre is considered reversible and upon occurrence of catastrophic post-operative complications, the vessel loop (occluding the PDV) can be readily removed.

As there was no PDV flow documented on Doppler ultrasound on Day 1 post-operatively, therapeutic anticoagulation was commenced to reduce the overall risks of portal venous thrombosis. Since the patient remained asymptomatic and the PDV remained occluded without any evidence of portal venous thrombosis at 3 months, it was thus considered that the second stage of the procedure for ligation and division of the PDV was no longer necessary.

**Figure 3:**
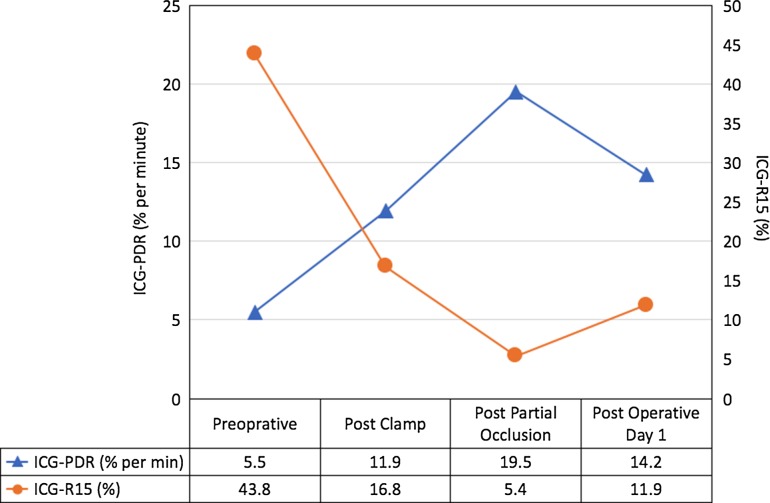
Perioperative measurements of indocyanine green (ICG) clearance is effective for the intra-operative evaluation of hepatic function. ICG-PDR, indocyanine green plasma disappearance rate; ICG-R15, indocyanine green retention ratio after 15 min.

Non-invasive measurements of ICG clearance is increasingly being used to assess hepatic function. In this case, we have also demonstrated the practicality of ICG clearance in the rapid intra-operative assessment of hepatic function after partial occlusion of the shunt (Fig. 3).

In conclusion, a minimally invasive laparoscopic approach is safe and feasible for the treatment of PDV. The consideration of a two-stage approach may be useful to manage PDV. In addition, the use of perioperative ICG clearances is feasible to rapidly evaluate the successful treatment of PDV.
